# Safety and efficacy of grazoprevir/elbasvir in the treatment of acute hepatitis C in hemodialysis patients

**DOI:** 10.1002/jmv.27374

**Published:** 2021-10-18

**Authors:** Qinghua Ji, Xudong Chu, Yugui Zhou, Xuan Liu, Wei Zhao, Wei Ye

**Affiliations:** ^1^ Department of Infectious Disease, School of Medicine Southeast University Nanjing Jiangsu China; ^2^ Department of Infectious Diseases The Affiliated Dongtai Hospital of Nantong University Dongtai Jiangsu China; ^3^ Department of Clinical Laboratory The Affiliated Dongtai Hospital of Nantong University Dongtai Jiangsu China; ^4^ Department of Clinical Laboratory The Second Hospital of Nanjing Nanjing Jiangsu China; ^5^ Department of Liver Disease The Second Hospital of Nanjing, Southeast University Nanjing Jiangsu China

**Keywords:** acute hepatitis C, efficacy, grazoprevir/elbasvir, hemodialysis‐dependent patients, safety

## Abstract

Treatment of hepatitis C virus (HCV) infection with direct‐acting antiviral agents (DAAs) in hemodialysis patients requires extensive consideration. At present, studies regarding DAAs for acute HCV infection in such patients are limited. The present study aims to evaluate the efficacy and safety of grazoprevir (GZR) plus elbasvir (EBR) treatment in acute hepatitis C (AHC) patients undergoing hemodialysis. Patients undergoing hemodialysis who had a nosocomial acute HCV infection were enrolled. All patients received GZR 100 mg/EBR 50 mg once daily for 12 weeks and were followed up for 12 weeks. Serum alanine transaminase (ALT), aspartate aminotransferase (AST), total bilirubin (TBIL), and HCV RNA levels were monitored during treatment and follow‐up periods. Sustained virologic response at 12 weeks after treatment cessation and treatment‐emergent adverse events (AEs) were assessed. A total of 68 AHC patients were enrolled. All patients were infected with HCV genotype 1b and achieved SVR12. Decreasing ALT, AST, and TBIL were observed over time in the first 4 weeks and became steady thereafter. Forty‐eight (70.59%) patients reported at least one AEs. The most common AEs were fatigue, headache, and nausea. Two AHC patients discontinued treatment due to serious but drug‐unrelated AEs. In conclusion, GZR/EBR has a high efficacy and safety profile in hemodialysis‐dependent patients with genotype 1b AHC.

## Highlights

1. All AHC patients undergoing hemodialysis achieved SVR12 after 12 weeks of GZR/EBR treatment.
2. Low baseline HCV RNA levels were related to early viral clearance during GZR/EBR treatment.
3. GZR/EBR treatment was tolerable and most patients had only mild AEs including fatigue, headache, and nausea during treatment.

## 1 INTRODUCTION

Hepatitis C virus (HCV) infection is associated with morbidity and mortality of patients with cirrhosis and hepatocellular carcinoma caused by chronic hepatitis C (CHC).^1^ The World Health Organization estimates that in 2015, there were 71 million people living with HCV infections worldwide, accounting for 1% of the global population, and there were 1.75 million new HCV infections diagnosed.^2^ In 2015, HCV infections were responsible for approximately 0.4 million deaths, mainly due to cirrhosis and hepatocellular carcinoma.^2^ HCV is transmitted primarily by parenteral routes, including unsafe healthcare practices in developing countries (unsterile healthcare injections, blood transfusions, and other invasive medical procedures) and intravenous drug use in developed countries.^1^ Patients undergoing hemodialysis are at high risk for HCV infection as they are commonly exposed to blood‐borne pathogens because of frequent intravenous access and catheter manipulation.^3^ The prevalence of HCV infection in hemodialysis‐dependent patients has been reported to be between 3.8% and 7.6%, while the data in China are 9.9% from the DOPPS study, which was dependent on economic development and is substantially higher than in the general population.^4^

Moreover, acute HCV infections in hemodialysis‐dependent patients are always silent and asymptomatic, and 65%–92% of the patients with acute hepatitis C (AHC) can develop CHC without treatment.^5^ HCV seropositive patients with advanced kidney disease may experience an increased risk of death and reduced access to renal transplantation.^6^, ^7^, ^8^ Cirrhosis is also a concern in such patients with long‐term HCV infection. Although the management of AHC has not reached a consensus, early treatment may be helpful to prevent chronic infection and avoid the risk factors that accelerate disease progression.

Until the introduction of direct‐acting antiviral agents (DAAs), the recommended treatment for HCV infection was a regimen of Peg‐interferon with ribavirin, which achieved a sustained virologic response (SVR) in 54.4%–87.0% of patients, even after optimization.^9^, ^10^ Hemodialysis was considered a contradiction of HCV treatment due to the severe side effects of Peg‐interferon and ribavirin before the DAA era. The development of DAAs in 2011 has revolutionized hepatitis C management.^11^ At present, available oral regimens are based on the combination of DAAs with or without ribavirin, which has excellent efficacy and safety profiles for most CHC patients according to clinical trials and real‐world studies.^1^, ^11^ However, data on the efficacy of DAAs in acute or recent HCV‐infected patients vary with different treatment regimens and durations. Several clinical trials and cohort studies were conducted to describe the optimal management for acute or recent HCV infection. Altogether, the regime containing sofosbuvir plus ribavirin showed suboptimal efficacy with the SVR12 rate ranging from 32% to 92% and it is not recommended in current guidelines,^12^, ^13^, ^14^ whereas the second generation DAA regimes had promising results. The DAHHS2 study showed that treatment of 80 patients with genotypes 1 and 4 AHC using grazoprevir (GZR) plus elbasvir (EBR) for 8 weeks can achieve an SVR12 rate of 99%.^15^ Martinello et al.^16^ also reported that treatment of 30 patients with recent HCV genotype 1 infection using ombitasvir/paritaprevir/ritonavir plus dasabuvir (ProD) for 8 weeks can achieve an SVR12 rate of 97%.^16^ Treatment of 20 patients with an acute HCV genotype 1 monoinfection using ledipasvir plus sofosbuvir resulted in an SVR12 rate of 100%.^17^ Another study also reported an SVR12 rate of 100% with interferon‐free therapy for AHC in V‐positive patients.^14^

The C‐SURFER study first investigated the efficacy and safety of EBR/GZR in 224 HCV genotype 1 infected CHC patients with Stage 4 or 5 chronic kidney disease (CKD). They found that the SVR12 was 99% with only one relapse 12 weeks after the end of treatment and the adverse events (AEs) (headache, nausea, and fatigue) were comparable to those in the placebo‐control group.^18^, ^19^ Several other Phase III trials and real‐world studies also reported comparable results in the same population with regimens containing protease inhibitors (PI) such as EBR/GZR, ProD, asunaprevir/daclatasvir, or glecaprevir/pibrentasvir.^20^, ^21^, ^22^, ^23^, ^24^, ^25^ However, studies on acute HCV‐infected patients undergoing hemodialysis are limited. One study in 19 patients reported that sofosbuvir‐containing regimens were effective and safe for the treatment of acute HCV in patients undergoing hemodialysis.^26^

In May 2019, nosocomial HCV infections among hemodialysis‐dependent patients occurred in Dongtai People's Hospital of Jiangsu Province, and 68 patients were diagnosed with AHC. We evaluated the efficacy and safety of GZR plus EBR treatment for 12 weeks in these patients.

## 2 MATERIALS AND METHODS

### 2.1 Participants

This retrospective study (No. ChiCTR2000034389) enrolled patients undergoing hemodialysis who had AHC because of nosocomial HCV infections due to medical negligence. The study was performed according to the Declaration of Helsinki principles and the protocol was approved by the local institutional Ethics Committee. Written consent was waived due to the retrospective nature of the study. An observation of EBR/GZR utilization in CHC patients with hemodialysis in the same period was also conducted.

Anti‐HCV and liver function were detected in all of the hemodialysis patients every 6 months. The HCV RNA will be detected further if the anti‐HCV was positive or the ALT was abnormal. The diagnosis of acute HCV infection was based on the finding of recent positive anti‐HCV or HCV RNA in patients with negative anti‐HCV and normal liver enzyme levels 6 months before. The chronic HCV infection was positive for anti‐HCV and HCV RNA for more than 6 months. Routine testing revealed the first acute hepatitis C patient at this blood purification center. Then all of the patients undergoing hemodialysis were screened for anti‐HCV and HCV RNA.

Baseline information including age, sex, baseline HCV RNA levels, genotype, and certain laboratory data was collected from the records.

### 2.2 GZR/EBR treatment

All patients received GZR 100 mg/EBR 50 mg once daily for 12 weeks and were followed up for an additional 12 weeks.

### 2.3 Assessments

Serum alanine transaminase (ALT), aspartate aminotransferase (AST), total bilirubin (TBIL), and HCV RNA levels were detected at baseline, during treatment at 1 week, 2 weeks, 4 weeks, and 12 weeks, as well as at the final follow‐up 12 weeks after the end of treatment (EOT). HCV RNA levels were quantified using real‐time polymerase chain reaction using the Roche Cobas Ampliprep/Cobas Taqman HCV test V.2.0 (Roche) with a lower detection limit of 15 IU/ml. Virologic response (VR) and SVR were defined as undetectable HCV RNA levels during the treatment and follow‐up period, respectively. The efficacy was evaluated using SVR12.

### 2.4 Adverse events (AEs)

Safety was assessed by monitoring the AEs. AEs of all patients were recorded during the 12‐week treatment. Common AEs in the present study included itching, fatigue, headache, nausea, vomiting, insomnia, dizziness, diarrhea, and so forth.

### 2.5 Statistical analysis

All data are presented as *n* (%) or mean ± standard deviation. To evaluate the efficacy analysis, a univariate analysis was performed using the *χ* ^2^ test, and factors with *p* values less than 0.1 were entered into the multivariate analysis, which was performed to identify independent prognostic factors. A *p* value less than 0.05 was considered statistically significant.

## 3 RESULTS

### 3.1 Baseline characteristics of participants

A total of 68 patients were identified with nosocomial HCV infections during the first quarter of 2019. The accurate date of exposure remained unknown but it was within 6 months due to their semiannual screening for the HCV antibody. After disease assessment, all the AHC patients received 12 weeks of treatment with EBR/GZR. Data was also collected for eleven CHC patients who received 12 weeks of EBR/GZR treatment in the same period. Baseline information at treatment initiation of all patients is shown in Table 1. There were 50 males (73.5%) in the AHC group and 10 males (90.9%) in the CHC group. The average ages were 55.6 and 54.1 years, respectively. All patients were infected with HCV genotype 1b. The proportions of patients with a high viral load (HVL ≥ 800 000 IU/ml) were 54.4% (37/68) and 27.3% (3/11) in the AHC and CHC groups, respectively. None of the patients in the CHC group had been diagnosed with cirrhosis.

**Table 1:** Patient characteristics at baseline

	AHC (*n* = 68)	CHC (*n* = 11)
Epidemiological characteristics		
Sex (male, %)	50 (73.5)	10 (90.9)
Age (years)	55.6 ± 11.5	54.1 ± 10.0
Hemodialysis (*n*, %)	68 (100)	11 (100)
HBV co‐infection	7 (10.3)	2 (18.2)
HCV infection parameters		
Genotype, 1b	68 (100)	11 (100)
HCV‐RNA (log IU/ml)	5.6 ± 1.4	5.1 ± 1.0
Anti‐HCV (+)	40 (58.8)	11 (100)
Laboratory parameters		
Hb (g/L)	104.1 ± 21.8	96.0 ± 15.1
PLT (×10^9^/L)	167.8 ± 54.2	149.0 ± 30.2
Albumin (g/L)	40.0 ± 5.0	38.4 ± 6.1
ALT (U/L)	69.6 ± 99.7	39.6 ± 38.2
AST (U/L)	36.8 ± 47.1	22.9 ± 15.8
Bilirubin (μM)	8.0 ± 5.6	7.2 ± 3.5
Urea nitrogen	23.8 ± 7.4	27.8 ± 12.1
Creatinine (μM)	943.8 ± 274.5	1130.3 ± 206.7

Abbreviations: AHC, acute hepatitis C; ALT, alanine transaminases; AST, aspartate aminotransferase; CHC, chronic hepatitis C; HBV, hepatitis B virus; HCV, hepatitis C virus; Hb, hemoglobin; PLT, platelet; TBIL, total bilirubin.

Additionally, seven patients in AHC and two patients in CHC were hepatitis B virus (HBV) surface antigen‐positive, and the HBV‐DNA levels were not detected at baseline. HBV reactivation, which is defined as detectable HBV‐DNA, was monitored in one AHC patient during treatment and two patients (one in AHC and one in CHC) after treatment cessation. All three patients received entecavir after HBV reactivation was diagnosed. Interestingly, only 58.8% (40/68) of AHC patients acquired anti‐HCV antibody seroconversion at baseline while all CHC patients were anti‐HCV positive. None of those AHC patients with negative anti‐HCV achieved seroconversion even after the follow‐up period.

### 3.2 Efficacy

**Figure 1:**
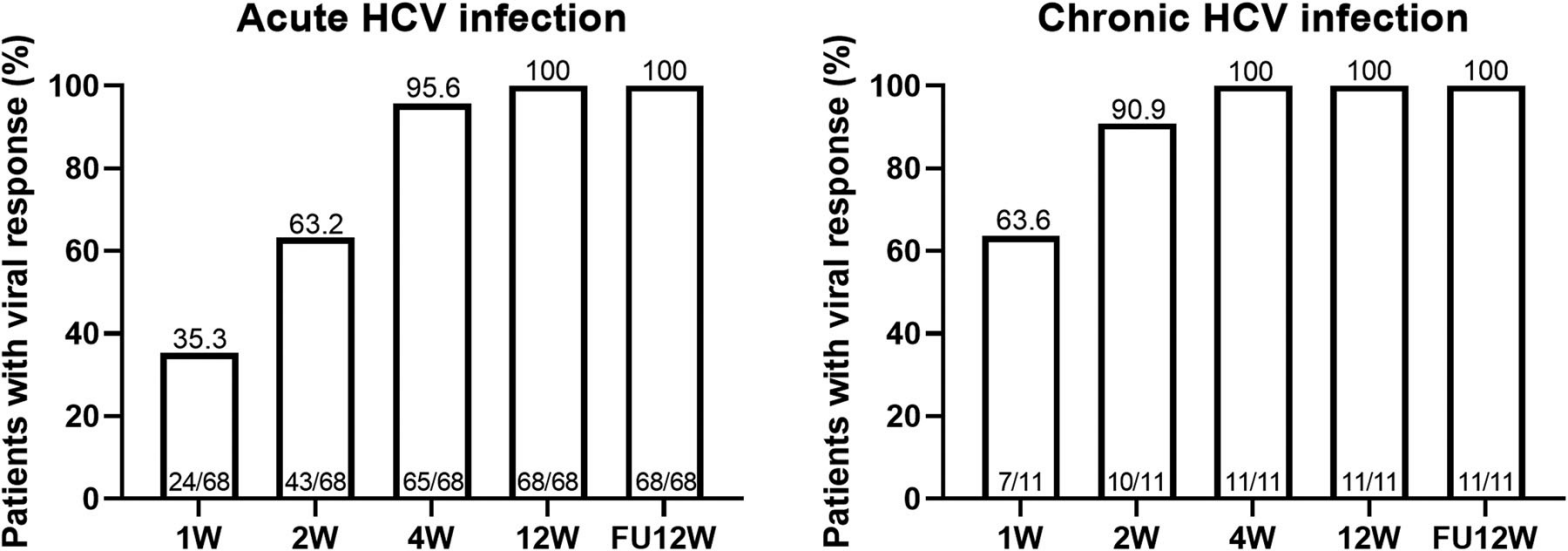
Virologic response during treatment and follow‐up. FU, follow‐up; HCV, hepatitis C virus

**Figure 2:**
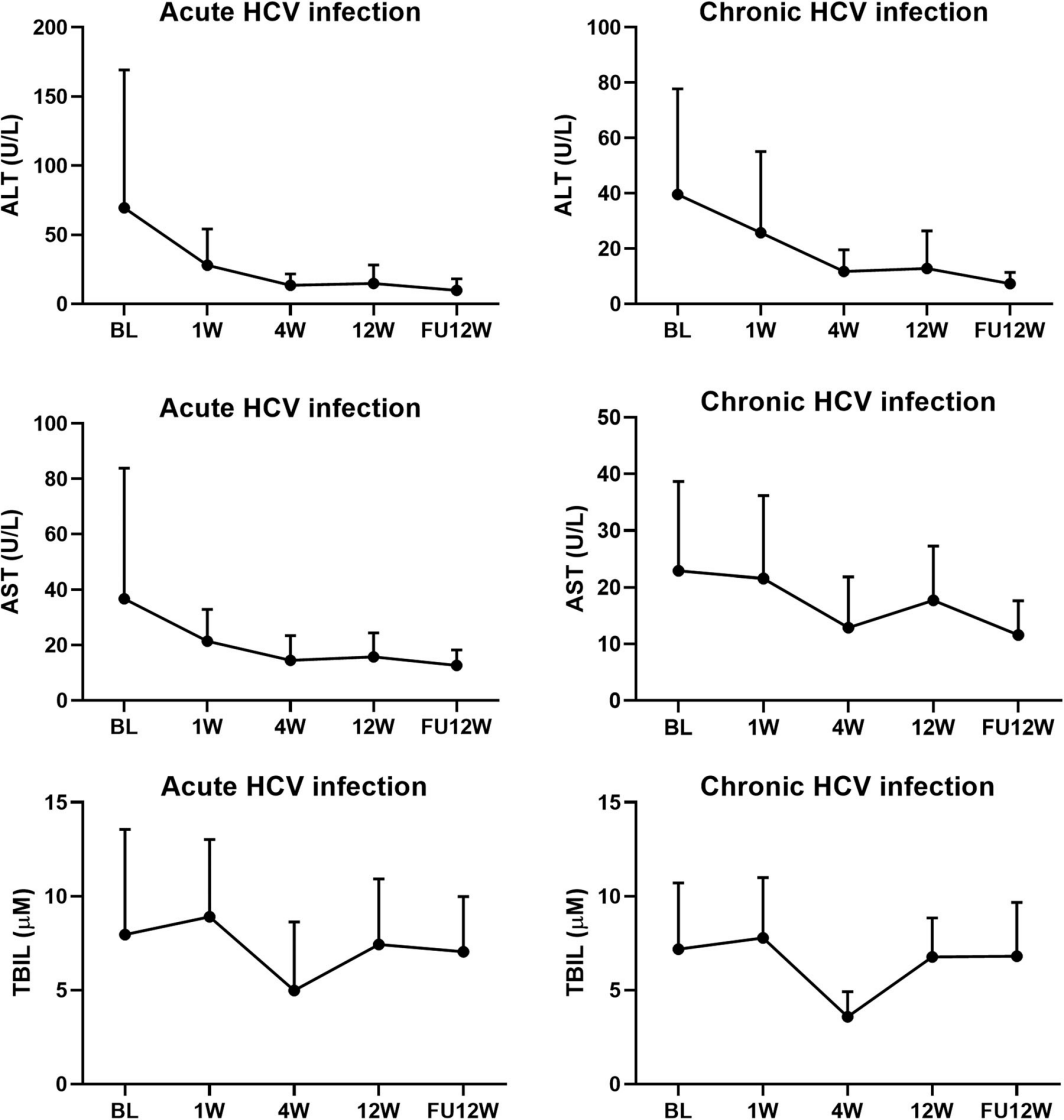
The dynamic change of ALT/AST and TBIL levels during treatment and follow‐up. ALT, alanine transaminases; AST, aspartate aminotransferase; BL, baseline; FU, follow‐up; HCV, hepatitis C virus; TBIL, total bilirubin

All patients achieved virologic responses at EOT and SVR12 both in the AHC and CHC groups, (Figure 1) and no patients were lost to follow‐up. All patients showed a decrease in ALT and AST levels over time within the first 4 weeks and normal levels were maintained thereafter (Figure 2).

Early virologic response was defined as undetected HCV‐RNA at treatment weeks 1, 2, and 4 (VR1, VR2, and VR4; Figure 1). At Week 4, almost all patients achieved viral eradication. All CHC patients (11/11) and 95.6% of AHC patients (65/68) achieved VR4. The risk factors that affected early virologic response were calculated in the AHC group, but those in the CHC group were not analyzed due to the small sample size. Early treatment viral kinetics were related to baseline HCV‐RNA levels. Patients with a high baseline viral load had significantly lower rates of VR1 and VR2 than those with a low baseline viral load (Table 2). Moreover, multivariate analysis showed baseline HCV levels can predict VR1 (Table 3). However, due to all patient with lower RNA level achieved VR2, the number of patients without VR2 was 0 in the lower group, multivariate analysis cannot be performed for VR2.

**Table 2:** Virologic response at the indicated time points

Baseline	Total (*n* = 68)	VR1 (*n* = 24）	*p* value	VR2 (*n* = 43)	*p* value	VR4 (*n* = 65)	*p* value
AST			0.568		0.744		0.196
Abnormal	17	7		11		15	
Normal	51	17		32		50	
ALT			0.573		0.744		0.056
Abnormal	25	10		16		22	
Normal	43	14		27		43	
TBIL			0.906		0.497		0.883
Abnormal	5	2		2		5	
Normal	63	22		41		60	
HCV RNA levels			<0.001		<0.001		0.241
logRNA ≥ 5.9	36	2		11		33	
logRNA < 5.9	32	22		32		32	

Abbreviations: ALT, alanine transaminases; AST, aspartate aminotransferase; HCV, hepatitis C virus; TBIL, total bilirubin.

**Table 3:** Multivariate analysis for VR1

	*p* value	OR (95%CI)
HCV RNA levels (high vs. low)	<0.001	37.400 (7.475–187.127)

Abbreviations: CI, confidence interval; HCV, hepatitis C virus; OR, odds ratio.

### 3.3 Safety

The safety profile of EBR/GZR is summarized in Table 4. There were 55 (70.59%) patients who reported a total of 113 AEs, with 48 AHC patients who reported 100 AEs and seven CHC patients who reported 13 AEs. The most common AE was fatigue (26.58%), followed by headache (18.99%), and nausea (18.99%). Drug‐related AEs were reported in 29 (36.71%) patients with a frequency of 50 total events. Two AHC patients discontinued treatment after 50 days and 44 days due to serious AEs, unrelated to the drugs (infective endocarditis and gastrointestinal bleeding, respectively). In addition, these two patients still achieved SVR12.

**Table 4:** Safety and adverse events (AEs) during the 3‐month treatment period

Events	AHC		CHC		Total	
	*n* = 68	%	*n* = 11	%	*n* = 79	%
Any AEs (person)	48	70.59	7	63.64	55	69.62
Any AEs (person × time)	98		13		111	
Any AEs (person × time, including SAEs)	100		13		113	
Itching	10	14.71	1	9.09	11	13.92
Fatigue	19	27.94	2	18.18	21	26.58
Headache	13	19.12	2	18.18	15	18.99
Nausea	12	17.65	3	27.27	15	18.99
Vomiting	8	11.76	1	9.09	9	11.39
Insomnia	11	16.18	1	9.09	12	15.19
Dizziness	4	5.88	0	0.00	4	5.06
Diarrhea	5	7.35	1	9.09	6	7.59
Drug‐related AEs (person)	26	38.24	3	27.27	29	36.71
Drug‐related AEs (person × time)	45	66.18	5	45.45	50	63.29
SAEs	2		0		2	
Drug‐related SAEs	0		0		0	
Discontinuation due to an AE	2		0		2	
	0				0	

Abbreviations: AHC, acute hepatitis C; CHC, chronic hepatitis C; SAE, severe adverse event.

The most common laboratory abnormality was mild and moderate ALT/AST elevation. In this study, elevated ALT and AST levels were observed in 23.5% (16/68) and 8.8% (6/68) of AHC patients, and 9.1% (1/11) and 18.2% (2/11) of CHC patients, respectively (Table 5). Only two patients in the AHC group had elevated TBIL levels during treatment. To be specific, 75% (12/16) of ALT elevation and 66.6% (4/6) of AST elevation occurred within the first 4 weeks during DAA treatment in the AHC group.

**Table 5:** Laboratory test results during the 3‐month treatment period

Abnormal laboratory test results	AHC		CHC	
	*n* = 68	%	*n* = 11	%
Grade 1 ALT elevation (1–3 × ULN)	14	20.59	1	9.09
Grade 2 ALT elevation (3–5 × ULN)	2	2.94	0	0
Grade 1 AST elevation (1–3 × ULN)	6	8.82	2	18.18
Grade 1 AST elevation (3–5 × ULN)	0	0	0	0
Grade 1 TBIL elevation (1–1.5 × ULN)	1	1.47	0	0
Grade 2 TBIL elevation (1.5–3 × ULN)	1	1.47	0	0

Abbreviations: AHC, acute hepatitis C; ALT, alanine transaminases; AST, aspartate aminotransferase; CHC, chronic hepatitis C; ULN, upper limit of normal; TBIL, total bilirubin.

Among those patients who experienced liver function abnormalities, the two patients that discontinued treatment early still had mildly elevated ALT levels and one other patient had abnormal TBIL levels at the 12‐week follow‐up.

## 4 DISCUSSION

Hemodialysis‐dependent patients are at a higher risk of HCV infection because of their potential exposure to blood‐borne pathogens through frequent intravenous access and catheter manipulation.^3^ This study is one of the few real‐world observational investigations on interferon‐free DAA utilization in hemodialysis‐dependent patients with acute genotype 1b HCV infection. All 68 patients acquired unexpected nosocomial HCV transmission. Previous reports indicate that a high proportion of hemodialysis patients who had acute HCV infections will develop chronic hepatitis and the incidence of spontaneous viral clearance is lower than that in the general population.^27^, ^28^ Early anti‐HCV treatment is an alternative option in such patients. In most cases, the anti‐HCV antibody can be detected 12 weeks after exposure.^29^ Interestingly, in the present study, anti‐HCV was detected in only 58% of acute patients even after the 12‐week follow‐up period. The prolonged seroconversion time was consistent with that reported in immuno‐compromised individuals.^30^, ^31^ In addition, elevated ALT/AST levels that represent liver injury were not very common in the early stage of acute HCV infection. Therefore, it would be a reasonable approach to assess HCV viral load with an HCV RNA quantitative test if there is reason to suspect infection.

If acute HCV infections are left untreated, the infection becomes persistent in 65%–92% of hemodialysis‐dependent patients.^5^ HCV infections can accelerate the decline in kidney function, worsen the prognosis of patients with pre‐existing CKDs, and adversely decrease the health‐related quality of life of patients with CKD combined with HCV infection.^32^ All‐cause cardiovascular morbidity and mortality also increase in hemodialysis‐dependent patients with HCV infection.^33^ Additionally, immediate treatment of acute HCV with DAAs can improve clinical outcomes and be highly cost‐effective compared with deferring treatment until the chronic phase of infection.^34^ Therefore, it is rational for hemodialysis‐dependent patients with AHC to receive treatment after diagnosis. The 2018 EASL guideline recommends patients with acute hepatitis C should be treated with DAA combinations for 8 weeks.^35^ The 2018 AASLD‐IDSA guideline does not recommend the exact treatment duration for acute HCV infection.^36^ However, both two guidelines recommend 12 weeks of GZR/EBR treatment for patients with end‐stage renal disease on hemodialysis.^35^, ^36^ Rockstroh et al.^37^ reported a compromised 77% SVR rate when SOF/LDV treatment was shortened in HIV patients with new HCV infections. Additionally, high baseline HCV‐RNA levels are an important risk factor associated with treatment failure following short‐duration DAA therapy.^12^, ^37^ A recent study showed that the SVR12 of an 8‐week course of daclatasvir and half‐dose SOF in acute hepatitis C patients with eGFR less than 30 ml/min was 25/27 (92.6%) and 25/26 (96.2%) on an intention‐to‐treat and per‐protocol basis, respectively.^38^ Taken together, the 12‐week regime may be the best option to treat newly HCV‐infected patients under hemodialysis. Therefore, a 12‐week duration of GZR/EBR was performed in the study.

In this study, we are the first to evaluate the efficacy and safety of GZR/EBR treatment for AHC and CHC in hemodialysis‐dependent patients. For the CHC group, this treatment consistently showed a high SVR rate that was comparable with published data from phase III clinical trials and real‐world data in the same population.^18^, ^23^, ^24^ DAA treatment of HCV infection had high effectiveness and safety in patients who undergo hemodialysis or kidney transplantation in the real‐world study. All patients achieved SVR and the creatinine concentration, eGFR, and proteinuria remained stable in the majority of patients.^25^ However, studies on acute HCV‐infected patients undergoing hemodialysis are limited, and our results have filled this gap in knowledge. All 68 acute patients achieved SVR12, which is comparable to the published data for EBR/GZR utilization in general AHC patients and CHC patients undergoing hemodialysis.^15^, ^18^, ^19^ With great consistency, no differences in SVR12 were observed regardless of patient age, sex, or baseline genotype 1b HCV‐RNA level. Thus, treatment with GZR/EBR, which has been previously shown to have excellent efficacy in general AHC patients as well as CHC patients undergoing hemodialysis, was applied in the present study to treat AHC patients undergoing hemodialysis. Furthermore, when analyzing the predictive factor for early treatment outcome, we found that low baseline HCV RNA levels were related to early viral clearance during treatment of HCV infections with GZR/EBR in hemodialysis‐dependent patients. This was consistent with previous reports, that baseline or early HCV RNA can predict prognosis or treatment outcomes during DAA treatment.^39^, ^40^ In this study, the difference in pharmacokinetics between the AHC and CHC groups at an early stage of treatment may be due to a higher proportion of patients with HVL in the AHC group. Although the baseline HCV‐RNA levels may affect the early virologic response, they had no impact on the final SVR12 result. Prior lower virologic response in the early treatment period may not predict a suboptimal efficacy even for those with high HCV‐RNA levels due to its high efficacy. Therefore, it may be reasonable to decrease the frequency of RNA quantitative tests and simplify the treatment cascade.

Our data also showed that most patients had only mild AEs during treatment. The most common AEs were fatigue, headache, and nausea. All AEs were tolerated, but two patients discontinued treatment due to serious AEs unrelated to the drugs, which were similar to previous reports in the C‐SUFER study and were comparable to those in the placebo control group.^19^ For patients with advanced CKD with or without hemodialysis, sofosbuvir‐based DAA regimes should be used very cautiously since renal clearance is the major pathway of elimination. EBR/GZR and other PI‐containing regimes are the first recommendations in such patients according to the guidelines.^35^, ^36^ However, PI‐containing regimes carry potential risks of liver toxicity. As such, treatment‐emergent liver injury is a major concern when using EBR/GZR therapy. We found that in the CHC group, only 9.09% (1/11) and 18.18% (2/11) patients experienced mild ALT or AST elevation, respectively. All treatment‐emergent ALT/AST elevations improved and were levels were restored to normal after cessation of treatment. The frequency of ALT/AST fluctuation in the AHC group was higher than that in the CHC group. We suspect that this may be caused by the disease progression of AHC. Most AHC patients also had restored ALT/AST levels after treatment, but the remaining ALT abnormalities in two patients who discontinued treatment early and achieved SVR12 still require further assessment. The inflammatory response triggered by HCV is a complicated procedure and the HCV infection is only the initiator of the pathophysiological processes, while persistent inflammatory cytokine storms caused by the interaction between the virus and host immune system exacerbate the progression of liver inflammation.^41^ Therefore, transaminase changes during anti‐HCV treatment are related to many factors. Additional studies should be performed to investigate the underlying mechanisms. Taken together, these data indicate that GZR/EBR is well‐tolerated for AHC and CHC patients undergoing hemodialysis.

There were some limitations in the present study. First, the suspected time of infection is unknown and the observation time after the diagnosis was insufficient for HCV‐RNA monitoring. We could not complete a picture of viral kinetics and the possibility of spontaneous HCV clearance in Chinese acute HCV‐infected patients, or identify the optimal time for an intervention. Second, the assessment of liver stiffness changes after the onset of AHC failed to be conducted and we could not validate the liver histological assessment in this unique situation. Further prospective studies are necessary to address such questions.

## 5 CONCLUSIONS

In conclusion, our data confirmed that 12 weeks of the EBR/GZR regimen is efficient and tolerable for the treatment of genotype 1b AHC in hemodialysis‐dependent patients. Our study provides evidence that supports the use of GZR plus EBR to treat acute HCV infection in patients undergoing hemodialysis.

## CONFLICT OF INTERESTS

The authors declare that there are no conflicts of interest.

## AUTHOR CONTRIBUTIONS

Qinghua Ji, Wei Zhao, and Wei Ye conceived, designed, or planned the study. Qinghua Ji, Xudong Chu, Yugui Zhou, Xuan Liu, and Wei Ye collected or assembled the data. Qinghua Ji Performed or supervised analyses. Qinghua Ji, Xuan Liu, Wei Zhao, and Wei Ye interpreted the results. Qinghua Ji wrote sections of the initial draft. Qinghua Ji, Xudong Chu, Yugui Zhou, Xuan Liu, Wei Zhao, and Wei Ye provided substantive suggestions for revision, reviewed and approved the final version of the article, and for all aspects of the work in ensuring that questions related to accuracy.

## Data Availability

The data underlying this article will be shared at reasonable request to the corresponding author.
